# Disseminated tuberculosis after Polatuzumab‐Vedotin based chemoimmunotherapy in a patient with Burkitt's lymphoma

**DOI:** 10.1002/ccr3.8838

**Published:** 2024-05-07

**Authors:** Sarah A. Elkourashy, Maria Benkhadra, Laila Shafei, Sulieman Abujarir, Rola Ghasoub

**Affiliations:** ^1^ Hematology Department National Center for Cancer Care and Research, Hamad Medical Corporation Doha Qatar; ^2^ Weill Cornell Medical College Doha Qatar; ^3^ Pharmacy Department National Center for Cancer Care and Research, Hamad Medical Corporation Doha Qatar; ^4^ Infectious Disease Department, Communicable Disease Center Hamad Medical Corporation Doha Qatar

**Keywords:** bendamustine, Burkitt's lymphoma, polatuzumab vedotin, reactivation, rituximab, tuberculosis

## Abstract

This report highlights the risk of latent tuberculosis (TB) reactivation after treatment with Polatuzumab Vedotin (PV), Rituximab, and Bendamustine (PBR protocol) despite appropriate chemoprophylaxis. A 48‐year‐old male with refractory Burkitt's lymphoma (BKL) was treated with PBR protocol. At baseline, the patient had a negative QuantiFERON test result, which turned out to be positive prior to starting PBR. He received chemoprophylaxis for 9 months and was compliant with treatment. One year later, he was admitted with COVID‐19 pneumonia and was treated according to the protocol. His symptoms persisted for 1 month. Investigations yielded disseminated TB‐infiltrated bone marrow and pleura. Downstream B‐cell and T‐cell depletion secondary to CD20 and CD79b antagonism may potentially explain the increased risk of TB reactivation associated with the combination of PV and rituximab. Further research is necessary to monitor the risk of TB reactivation among patients receiving a combination of PV and rituximab, especially in endemic areas with high prevalence and incidence of TB.

## 1 INTRODUCTION

Polatuzumab‐Vedotin (PV) is an antibody‐drug conjugate targeting CD79b that is approved for relapsed/refractory (R/R) diffuse large B‐cell lymphoma (DLBCL).^1^ PV is distinguished by its dual activity as an anti‐CD79b monoclonal antibody and a cytotoxic agent.^2^ It has been shown to possess the ability to halt CD79b activity by forming a covalent bond through a cleavable linker with the microtubule‐disrupting antimitotic agent monomethyl auristatin (MMAE)^2^, ^3^ upon internalization of PV into cancerous cells. MMAE suppresses cell division and induces apoptosis.^2^ CD79 is a heterodimer that plays a pivotal role in signaling transmission between B cells and B cell neoplasms. Therefore, CD79b was identified as the optimal therapeutic target for the treatment of B‐cell malignancies.^3^ Subsequently, the FDA approved the use of PV in the treatment of DLBCL in combination with bendamustine plus rituximab (PBR) and in the treatment of DLBCL in previously untreated patients in combination with rituximab, cyclophosphamide, doxorubicin, and prednisone (RCHOP).^4^, ^5^, ^6^ The most reported toxicities of PV include neutropenia, thrombocytopenia, anemia, and peripheral neuropathy.^5^ PV was not tested in patients with Burkitt's lymphoma (BKL). However, in a preclinical investigation, PV was found to exert tumor‐suppressive effects on human BKL‐derived B cells.^7^ Because there is limited data on how to manage patients with relapsed or resistant BKL, institutions may choose to use other regimens based on peer‐reviewed evidence when other treatment modalities failed. Alanzai et al. (2023) previously described a case of refractory BKL treated with Pola‐BR with a progression‐free survival (PFS) of more than 1 year.^8^ Here, we report this case subsequently, 1 year after the completion of his treatment for a unique clinical presentation of disseminated tuberculosis despite TB chemoprophylaxis, and we share our experience with his challenging management.

## 2 CASE REPORT

### 2.1 Case history and physical examination

**FIGURE 1:**
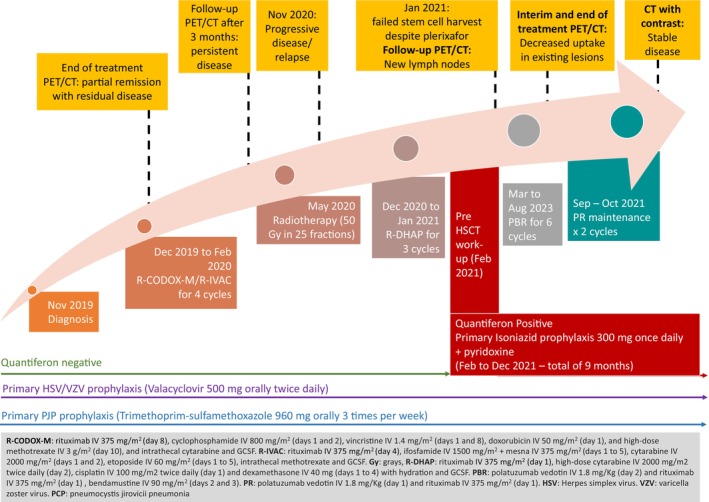
Treatment history.

A 48‐year‐old Filipino male with stage II bulky disease, BKL, was diagnosed in 2019. His full diagnosis history was previously reported by Alanzai et al. (2023). As shown in Figure 1, in 2021, the patient was admitted for stem cell mobilization after salvage chemoimmunotherapy and radiotherapy. His pre‐transplant workup showed a positive QuantiFERON result (latent TB), which was previously negative at the initial diagnosis. High‐resolution computed tomography (HRCT) did not show evidence of active lung disease. Subsequently, chemoprophylaxis with isoniazid (INH) 300 mg once daily and pyridoxine was initiated. Unfortunately, the stem cell mobilization attempt failed, but INH + pyridoxine was completed within 9 months.

As the patient was refractory to two lines of chemoimmunotherapy, the final recommendation was to start a PV‐based regimen as a case study to control the disease and prolong survival. The patient consented and started on the PV (1.8 mg/kg), bendamustine (90 mg/m^2^) and rituximab (375 mg/m^2^) (PBR) protocol. The patient tolerated the treatment well and maintained a good performance, apart from prolonged neutropenia secondary to bendamustine. After completing six cycles of PBR treatment, a second fluorodeoxyglucose (FDG)‐positron emission tomography (PET) scan was performed, and the results revealed further declining uptake in the prior lesions, with no new lesions (MTV 2.2 cm^3^). Two additional doses of PV and Rituximab were administered and completed by 2021. Three months after treatment completion, a follow‐up CT scan showed a decrease in the size of the right‐sided cervical masses and lymph node. No further complications were encountered.

### 2.2 Methods

A year later, the patient was admitted with fever, a history of dry cough, generalized fatigability, shortness of breath (SOB), and unintentional weight loss (10 kg) in the past month. Physical examination results were normal, except for a right‐sided lower chest zone crepitation. Laboratory tests showed leukopenia with a white blood cell (WBC) count of 0.3 × 10^3^/μL, absolute neutrophil count (ANC) 0.2 × 10^3^/μL, thrombocytopenia with a platelet count of 26 × 10^3^/μL, and anemia (hemoglobin of 7.9 g/dL). Chest radiography showed bilateral accentuation of lung reticulation and interstitial markings, with faint infiltrates scattered mainly in the left mid‐ and lower‐lung zones. His inflammatory markers were high: C‐reactive protein (CRP), 263 mg/L and procalcitonin 5.37 ng/mL. Blood cultures were sent and the patient was started on empirical antibiotics for febrile neutropenia.

COVID‐19 PCR showed positive results, with a CT value of 24.84. The patient was tachycardic, tachypneic, hypotensive, and hypoxic on 4 L of O2; therefore, he was treated as per the COVID‐19 protocol. Since his PET‐CT was normal 1 month ago, his pancytopenia was attributed to COVID‐19. His total WBC and neutrophil counts improved with granulocyte colony‐stimulating factor (G‐CSF), and the patient was discharged.

One month later, the patient was readmitted to the emergency department with SOB. Two weeks prior to presentation, the patient noted increasing fatigue, SOB on exertion, weight loss, and dry cough, which persisted since his last admission for COVID‐19. The patient reported that his wife had recently returned from travel and had no contact with sick individuals. The patient had a documented fever (38°C) in the hospital and no other major complaints. Initial laboratory examination revealed a hemoglobin of 5.2 g/dL, WBC count of 0.3 x 10^3^/μL, platelet count of 26 x 10^3^/μL; retic count of 21.9 x 10^3^/μL, prothrombin time/international normalized ratio (PT/INR) of 16.7 s/1.5, activated partial thromboplastin time (aPPT) of 28.9 s, lactate dehydrogenase (LDH) of 308 U/L, ferritin of 15,234.0 μg/L, total bilirubin of 0.23 μmol/L, aspartate aminotransferase (AST) of 43 U/L, alanine aminotransferase (ALT) of 69 U/L, and creatinine level of 81 μmol/L. His inflammatory markers revealed CRP of 172.6 mg/L, lactic acid of 4.8 mmol/L, and procalcitonin levels were 2.71 ng/mL.

Chest radiography on admission showed faint patchy and linear opacities in the left middle and lower lung fields, and right‐sided pleural effusion. The patient was found to be positive for the COVID antigen and was started on empirical antibiotics accordingly. The next day, the patient still had spiking fever; therefore, dexamethasone 6 mg was added as part of the COVID‐19 protocol. Additional investigations were ordered by the team, as the patient still had a spiking fever. His interleukin (IL)‐6 level was 302 pg/mL, and a bone marrow (BM) biopsy and aspiration were performed. Differential diagnoses based on his initial presentation included disease relapse, viral pneumonia, and macrophage activation syndrome. On day 6 of admission, the patient was transferred to the Medical Intensive Care Unit (MICU) because he was febrile and tachycardic with hypoxia. The patient was maintained on blood transfusion and broad‐spectrum antibiotic therapy.

**FIGURE 2:**
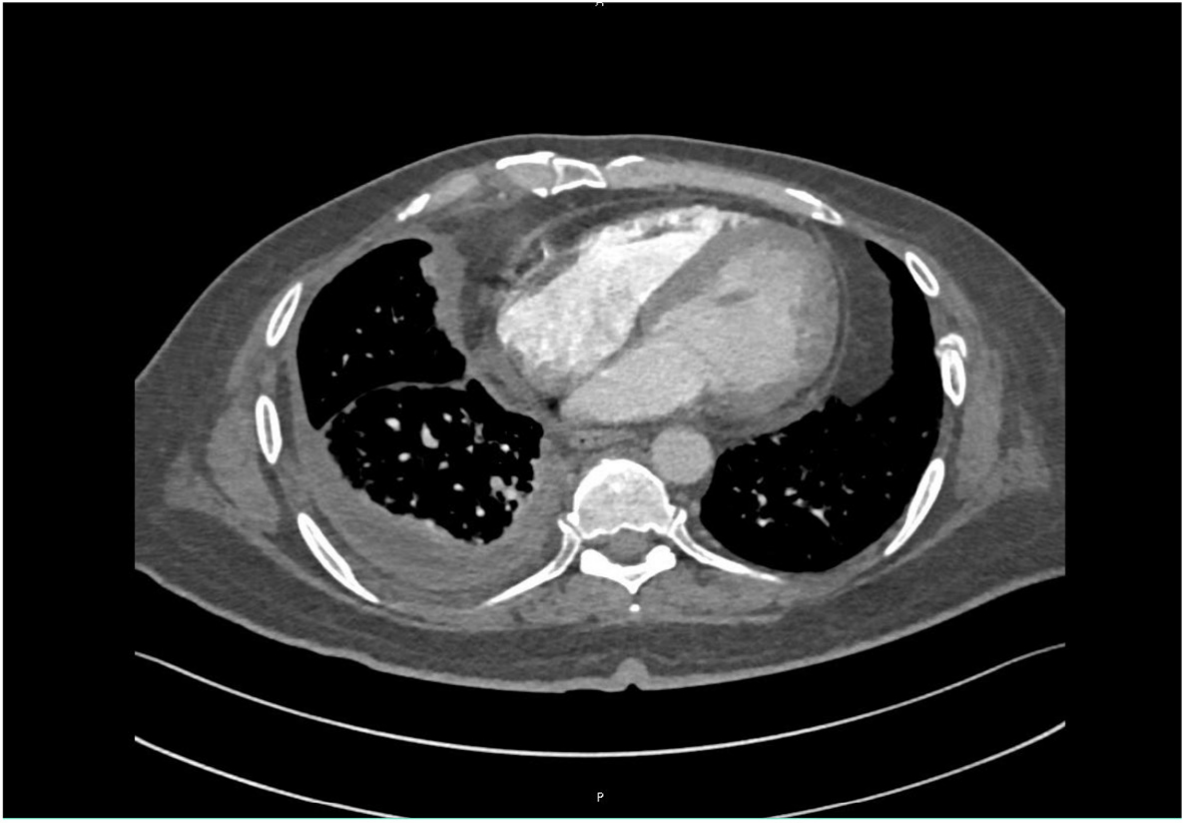
Right‐sided lower loculated pleural effusion with enhancing circumferential nodular thickening of the pleural wall.

**FIGURE 3:**
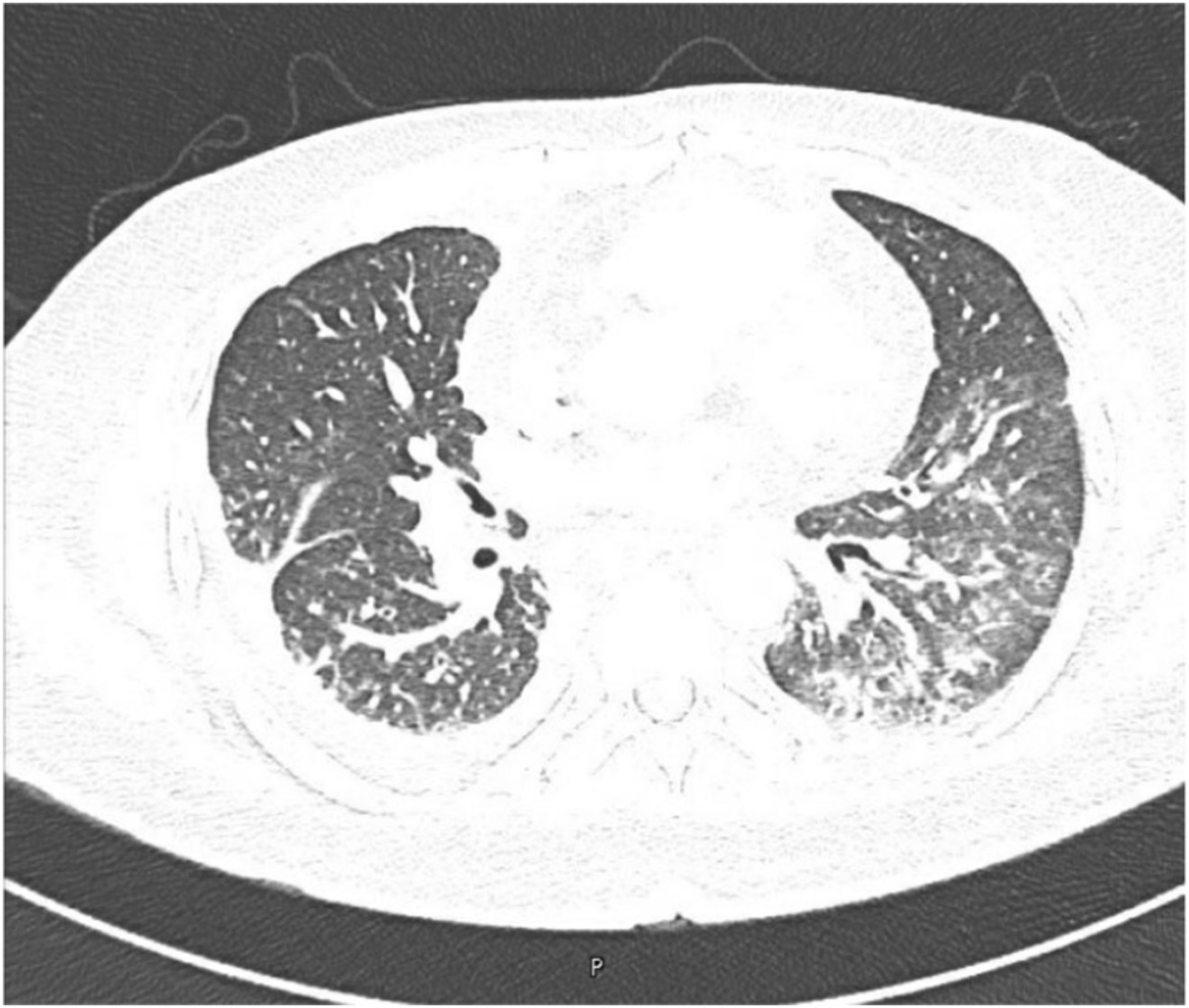
Diffuse left lower lung lobe peribronchovascular patchy ground glass opacities, along with diffuse intralobular septal wall thickening in the lower lung lobes bilaterally.

Ultrasound (US) of the thorax and pleural cavity showed a consolidated right lung with multiseptate cystic changes and right‐sided pleural effusion with an estimated volume of 16 × 12 × 13 cm (1267 cc) noted in the semi‐sitting position. Thoracic ultrasonography and diagnostic pleural tap were recommended, but the patient refused. Subsequently, a computed tomography (CT) pulmonary angiogram showed lung parenchymal changes suggestive of an infectious process and a right lower pleural effusion with a thick, nodular‐enhancing pleural wall. (Figures 2 and 3) At that time, the patient refused bronchoscopy or pleural tap. Additional workup was performed (Table 1), and an AFB smear and TB culture were sent.

**TABLE 1:** Additional workup.

Variable	Value	Normal range
Hepatitis B surface antibody	Negative	
Hepatitis B surface antigen	Negative	
Hepatitis B total core antibody	Negative	
Hepatitis C antibody	Negative	
HIV Ag/Ab combo	Negative	
Aspergillus galactomannan antigen	Negative	
Cytomegalovirus PCR	Negative	
Epstein–Barr virus PCR	Negative	
Rheumatoid factor	11 IU/mL	Normal high 14
ANA	Negative	
ANCA	Negative	

Abbreviations: ANA, antinuclear antibody; ANCA, antineutrophil cytoplasmic antibodies; HIV Ag/Ab, human immunodeficiency virus antigen/antibody; PCR, polymerase chain reaction.

### 2.3 Conclusion and results

**FIGURE 4:**
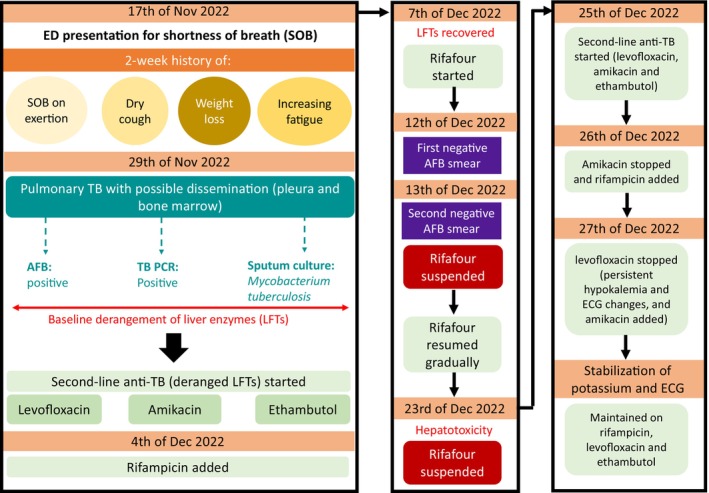
TB treatment history.

The BM results showed hypocellular bone marrow with decreased trilineage hematopoiesis, increased histiocytes, and multiple granulomas. There was no evidence of bone marrow involvement by BKL. The AFB smear and TB PCR results were positive (rifampicin sensitive). The patient was diagnosed with disseminated TB (involving the lungs, pleura, and bone marrow). Antibiotics were discontinued, and the patient was started on second‐line antitubercular treatment (ATT) because he had deranged LFTs. His TB treatment was challenging (Figure 4).

#### 2.3.1 Immunology

The patient was evaluated on the January 2023 by an immunologist for lasting immunosuppression after treatment. As shown in Table 2, the immunology workup on the 25th of December 2022 patient had a reduced CD3‐absolute count, CD3+/CD4+ absolute count and percentage, CD19, and CD3−/CD16 + CD56+ absolute counts. The immunologist determined that the patient had secondary immunodeficiency due to a history of lymphoma or rituximab therapy.

**TABLE 2:** Immunology workup.

Variable	Date	Value	Normal range	Value Interpretation
CD3	25/12/2022	78.9%	57.52–83.11	Normal
CD3 Abs Count	25/12/2022	234 cell/μL	856–2669	Low
CD3+/CD4+ %	25/12/2022	20.5%	31.45–62.38	Low
CD3+/CD4+ Abs count	25/12/2022	61 cells/μL	491–1734	Low
CD3+/CD8+ %	25/12/2022	54.6%	9.55–38.32	High
CD3+/CD8+ Abs count	25/12/2022	162 cells/μL	162–1074	Normal
CD19	25/12/2022	0%	5.89–24.21	Low
CD19 Abs Count	25/12/2022	0 cells/μL	73–562	Low
CD3‐/CD16+/CD56+ %	25/12/2022	20.6%	5.17–30.36	Normal
CD3‐/CD16+/CD56+ Abs count	25/12/2022	61 cells/μL	108–680	Low
CD4:CD8 Ratio	25/12/2022	0.4%	NA	

## 3 DISCUSSION

In our report, TB reactivation was observed in our patient despite administration of a prophylactic regimen. Several factors can influence the reactivation of TB in patients. These factors include immunosuppression and myelosuppression resulting from anti‐TNF‐α drugs, leading to B‐cell downstream activation and T‐cell depletion.^9^, ^10^ Considering the mechanism of action of PV, anti‐CD79b activity can lead to B‐cell depletion, which in turn leads to a cascade of downstream mechanisms such as altered cytokine release, leukocyte infiltration, and decreased T‐cell activity.^11^ Although anti‐CD79b was found to result in a lower level of B‐ cell depletion than anti‐CD20,^11^ the case in our patient was different, as he was administered polatuzumab and rituximab together, highlighting the fact that our patient was subjected to both anti‐CD79b and anti‐CD20 agents.

Rituximab, a monoclonal antibody that exerts an inhibitory effect on CD20, binds to the B‐cell surface and activates a complement‐dependent B‐cell cytotoxic effect, mediating a cytotoxic effect through anti‐body‐dependent cellular toxicity.^12^ It is worth mentioning that multiple infusions of anti‐CD20 agents perturbed the development of germinal centers and the spleen.^12^ Thus, B‐cell functions progressively diminish over time, leading to impairment of antigen presentation, antibody formation, and production.^13^ Therefore, reducing humoral immunity through the reduction of antibodies such as IgA, IgM, and IgG^13^ indicates the risk of TB reactivation after receiving rituximab.

The risk of TB reactivation increases in patients administered biological agents. A Cochrane review reported that 20 out of 10,000 patients on biological agents developed TB compared to four out of 10,000 patients who were on placebo.^14^ Jung and colleagues reported a case of a 63‐year‐old Caucasian female patient diagnosed with rheumatoid arthritis and a history of TB infection.^15^ The patient developed TB followed by chronic disseminated pulmonary aspergillosis after receiving rituximab, steroids, and methotrexate.^15^ In contrast, two other case reports pointed out that rituximab seemed to be a better option in the treatment of rheumatoid arthritis in patients who developed disseminated TB infection after receiving infliximab.^16^, ^17^

Several explanations can be proposed to better understand the risk of TB reactivation after the use of both PV and rituximab. Initially, the patient was immunodeficient following rituximab administration. Although a retrospective cohort study reported that the risk of TB reactivation associated with rituximab therapy was relatively low,^18^ it is still important to evaluate the risk of reactivation, especially during the concurrent administration of rituximab and PV. In addition, it could be inferred that the co‐administration of both mAbs led to the severe depletion of B cells, which led to an increased risk of TB reactivation. In the POLARIX trial, approximately 90% of patients who received PV with R‐CHOP had grade 3–4 lymphopenia and neutropenia. Therefore, these patients were offered primary prophylaxis with G‐CSF.^2^, ^6^

In addition to biological agents, TB reactivation can be significantly affected by other factors. For instance, patients diagnosed with hematologic cancers and solid tumors were found to have an increased risk of TB reactivation.^19^ According to a cross‐sectional study conducted in Saudi Arabia, patients receiving chemotherapy have a higher prevalence of TB than the general population.^20^ This is more common among patients diagnosed with solid tumors than among those with hematological cancers.^20^ Kim et al. reported that patients with solid malignancies had a 4.5‐times higher risk of TB infection than healthy individuals.^21^ In 2018, a study in Qatar found that approximately 5.58% of 215 patients with acute myelocytic leukemia were coinfected with TB.^22^

It is also important to note that our patient was infected with COVID‐19 before TB reactivation. The COVID‐19 virus was found to lead to multiple histological damages to the lungs, and it could play a significant role in TB reactivation secondary to immunosuppression due to the use of corticosteroids as a course of treatment.^23^ COVID‐19 plays a role in provoking a cytokine storm and exhaustion of T cell lymphocytes.^23^ A previous case report reported that two patients, aged 68 and 58 years, were subjected to TB reactivation after COVID‐19 infection.^24^ It has been highlighted that post‐COVID‐19 tuberculosis signs and symptoms may be neglected because of similarities in clinical presentations.^24^ Also, COVID‐19 treatment protocols expose patients to the risk of TB reactivation due to the excessive use of corticosteroids.^23^, ^24^ This lasting immunosuppression was evident in this patient's immunology workup (15 months after the last PBR), which showed persistent B‐cell and T‐cell depletion. However, this workup cannot provide a definite answer as to whether this persistent immunosuppression is the result of rituximab, PV, their combination, disease nature, heavy pre‐treatment, or COVID‐19 infection.

Even though our patient received TB prophylaxis for a total of 9 months, as recommended by the Centers for Disease Control and Prevention (CDC) guidelines, TB reactivation occurred.^14^ Similar protection rates were observed in patients receiving tuberculosis prophylaxis for 6 months compared to 12 months in several studies, and no increased protection was found in patients receiving prophylaxis for longer than 12 months.^15^ It has been reported that prophylaxis should be extended for up to 12 months in patients at high risk of TB reactivation. However, as the likelihood of safety concerns increases, a tradeoff between benefits and risks should be made.^15^

## 4 CONCLUSION

To our knowledge, this is the first case report of disseminated TB following PV‐chemoimmunotherapy. Mortality from chemoimmunotherapy‐associated TB may be efficiently reduced through increased awareness. Therefore, it is legitimate to monitor patients considered to be at high risk, for example, patients diagnosed with hematological malignancies who receive more than one monoclonal antibody, resulting in the depletion of B cell activity. This is particularly important for patients receiving both PV and rituximab in a single protocol, as the risk of immunodeficiency increases when these two agents are co‐administered. Further research is necessary to monitor the risk of TB reactivation among patients receiving a combination of PV and rituximab, especially in endemic areas with a high prevalence and incidence of TB.

## AUTHOR CONTRIBUTIONS

**Sarah A. Elkourashy:** Writing – review and editing. **Maria Benkhadra:** Writing – review and editing. **Laila Shafei:** Writing – original draft. **Sulieman Abujarir:** Writing – review and editing. **Rola Ghasoub:** Writing – original draft.

## FUNDING INFORMATION

Open Access funding provided by the Qatar National Library. The authors declare that they have received no financial support in relation to this report.

## ETHICS STATEMENT

The case report was approved by the Hamad Medical Corporation's Medical Research Center under the Number (MRC‐04‐23‐570).

## CONSENT

Written informed consent was obtained from the patient to publish this report in accordance with the journal's patient consent policy.

## Data Availability

The data that support the findings of this study are not publicly available due to privacy reasons but are available on reasonable request from corresponding author.
